# A novel intra-operative, high-resolution atrial mapping approach

**DOI:** 10.1007/s10840-015-0061-x

**Published:** 2015-10-24

**Authors:** Ameeta Yaksh, Lisette JME van der Does, Charles Kik, Paul Knops, Frans BS Oei, Pieter C van de Woestijne, Jos A Bekkers, Ad JJC Bogers, Maurits A Allessie, Natasja MS de Groot

**Affiliations:** Department of Cardiology, Erasmus Medical Center, PO Box 2040, ‘s Gravendijkwal 230, 3000 CA Rotterdam, The Netherlands; Department of Physiology, Cardiovascular Research Institute Maastricht, Maastricht, The Netherlands; Department of Cardiothoracic Surgery, Erasmus Medical Center, Rotterdam, The Netherlands

**Keywords:** Electrophysiology, Atrial fibrillation, Epicardial mapping technique, Technical report

## Abstract

**Purpose:**

A new technique is demonstrated for extensive high-resolution intra-operative atrial mapping that will facilitate the localization of atrial fibrillation (AF) sources and identification of the substrate perpetuating AF.

**Methods:**

Prior to the start of extra-corporal circulation, a 8 × 24-electrode array (2-mm inter-electrode distance) is placed subsequently on all the right and left epicardial atrial sites, including Bachmann’s bundle, for recording of unipolar electrograms during sinus rhythm and (induced) AF. AF is induced by high-frequency pacing at the right atrial free wall. A pacemaker wire stitched to the right atrium serves as a reference signal. The indifferent pole is connected to a steal wire fixed to subcutaneous tissue. Electrograms are recorded by a computerized mapping system and, after amplification (gain 1000), filtering (bandwidth 0.5–400 Hz), sampling (1 kHz) and analogue to digital conversion (16 bits), automatically stored on hard disk. During the mapping procedure, real-time visualization secures electrogram quality. Analysis will be performed offline.

**Results:**

This technique was performed in 168 patients of 18 years and older, with coronary and/or structural heart disease, with or without AF, electively scheduled for cardiac surgery and a ventricular ejection fraction above 40 %. The mean duration of the entire mapping procedure including preparation time was 9 ± 2 min. Complications related to the mapping procedure during or after cardiac surgery were not observed.

**Conclusions:**

We introduce the first epicardial atrial mapping approach with a high resolution of ≥1728 recording sites which can be performed in a procedure time of only 9±2 mins. This mapping technique can potentially identify areas responsible for initiation and persistence of AF and hopefully can individualize both diagnosis and therapy of AF.

## Introduction

Atrial fibrillation (AF) can be eliminated by ablation of either the trigger or the substrate perpetuating AF. A multi-site, high-resolution mapping approach of the entire atria which can, beside localizing sources generating AF in patients with trigger-driven AF, identify the substrate responsible for perpetuation of AF would be desirable. Such a mapping approach would allow individualization of the diagnosis of AF and subsequently also of AF therapy which is at present not available. In this report, we introduce a novel high-resolution, multi-site epicardial mapping technique of the entire atria as a routine procedure during open chest surgery aiming for identification of the arrhythmogenic substrate underlying AF.

## Material and methods

### Surgical technique

**Fig. 1:**
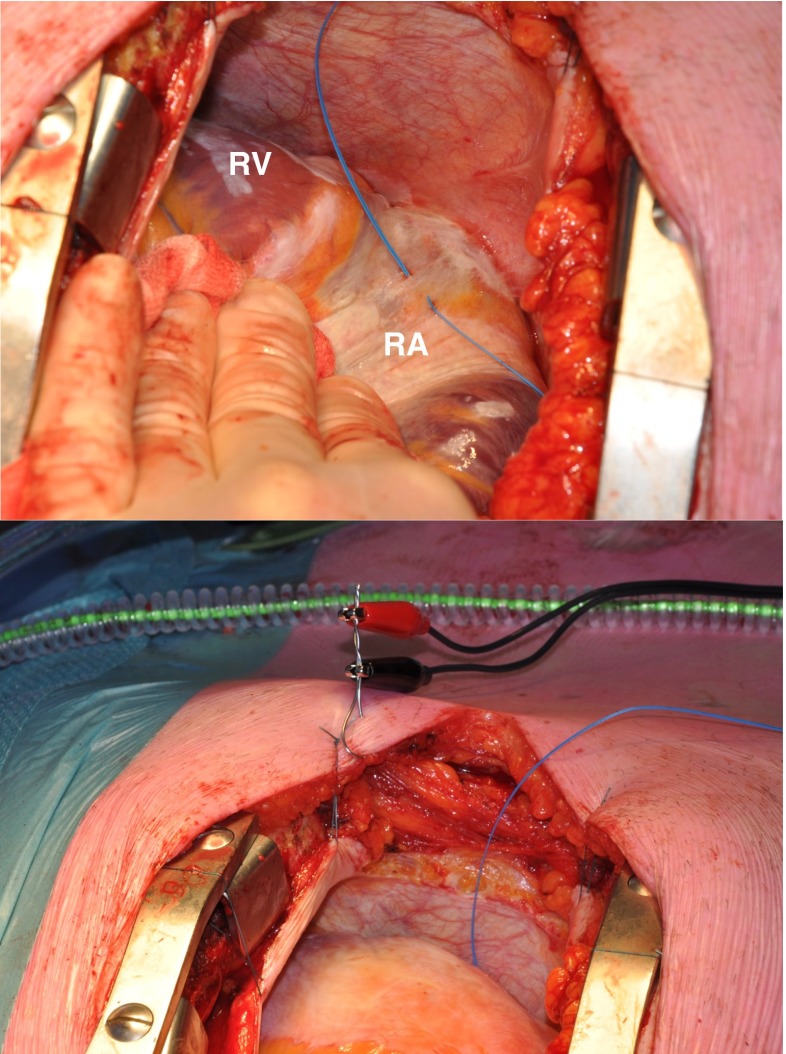
A pacemaker wire stitched to the right atrial free wall serving as a temporal reference electrode (*top*). A steal wire fixed to (sub)cutaneous tissue serving as the indifferent electrode (*bottom*). *RA* right atrium, *RV* right ventricle

**Fig. 2:**
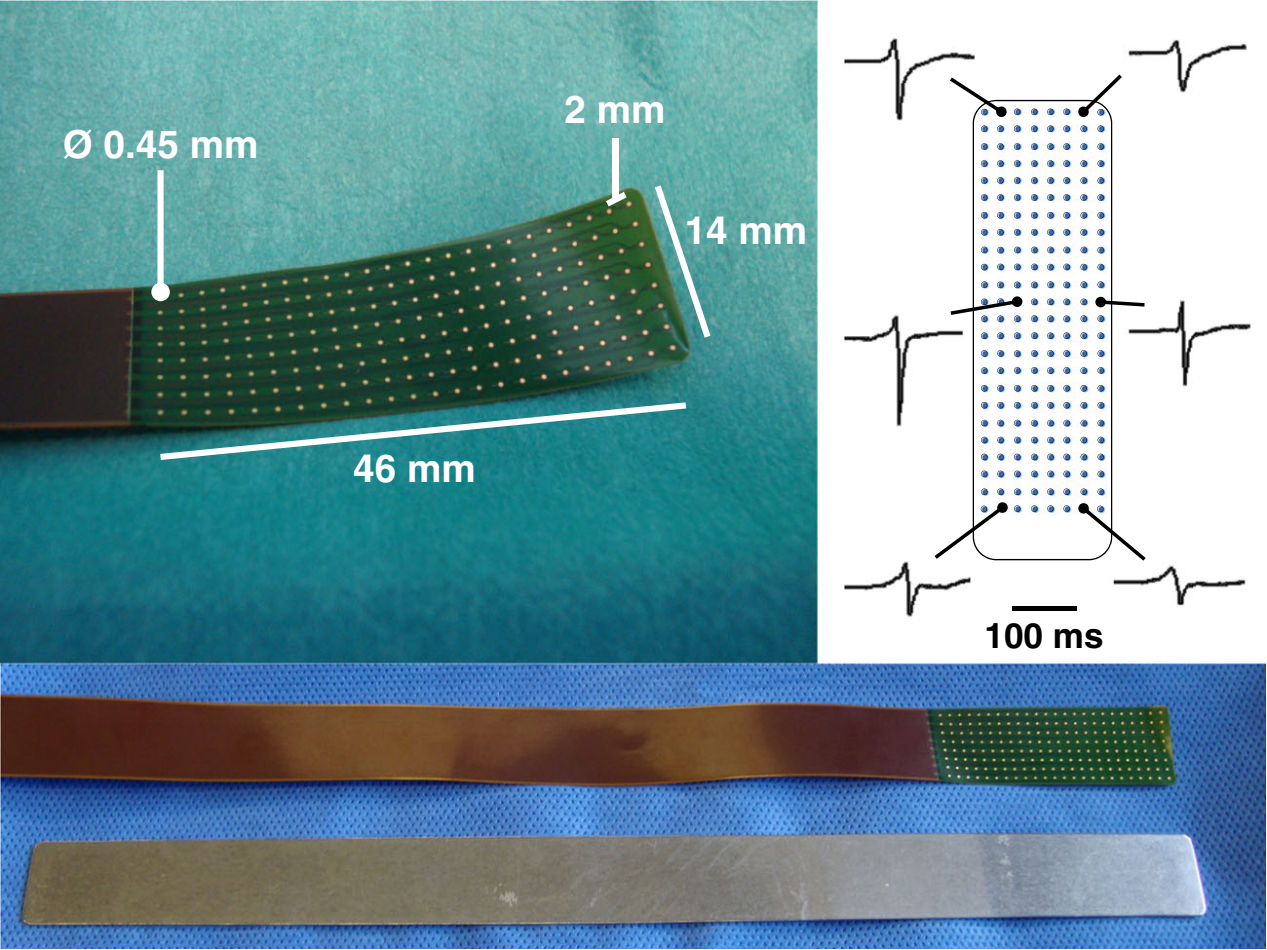
Mapping array containing 192 unipolar electrodes (*top left*). Examples of recorded electrograms at proximal, middle and distal electrodes of the array (*top right*). Mapping array and the identically shaped steel spatula (*bottom*)

Prior to commencement to extra-corporal circulation, after heparinization and arterial cannulation, a temporary bipolar epicardial pacemaker wire is stitched to the right atrial free wall serving as a temporal reference electrode. The indifferent electrode consists of a steal wire fixed to subcutaneous tissue of the thoracic cavity (Fig. 1). Epicardial mapping during sinus rhythm and (induced) AF is performed with a custom-made flexible 192-unipolar electrode mapping array, mounted on a custom-made spatula if preferred by the surgeon. The spatula can be bended to match the atrial curvature (Fig. 2). If AF is not the presenting rhythm, AF is induced by fixed rate pacing at the right atrial free wall using a different temporary bipolar pacing wire. Recordings of real-time epicardial electrograms from Bachmann’s bundle are used to confirm atrial capture. Fixed rate pacing is started at a rate of 200 beats per minute (bpm). If an AF induction attempt is not successful after three burst attempts, the rate is increased by 50 bpm, up to maximal 400 bpm until AF occurs or atrial refractoriness is reached. After completion of the mapping procedure, AF is terminated by electrical cardioversion or sustained until cardioplegia is conducted, depending on the operators’ preference. In case of AF as the initial heart rhythm, electrical cardioversion is performed in order to map sinus rhythm after completing mapping of AF.

**Fig. 3:**
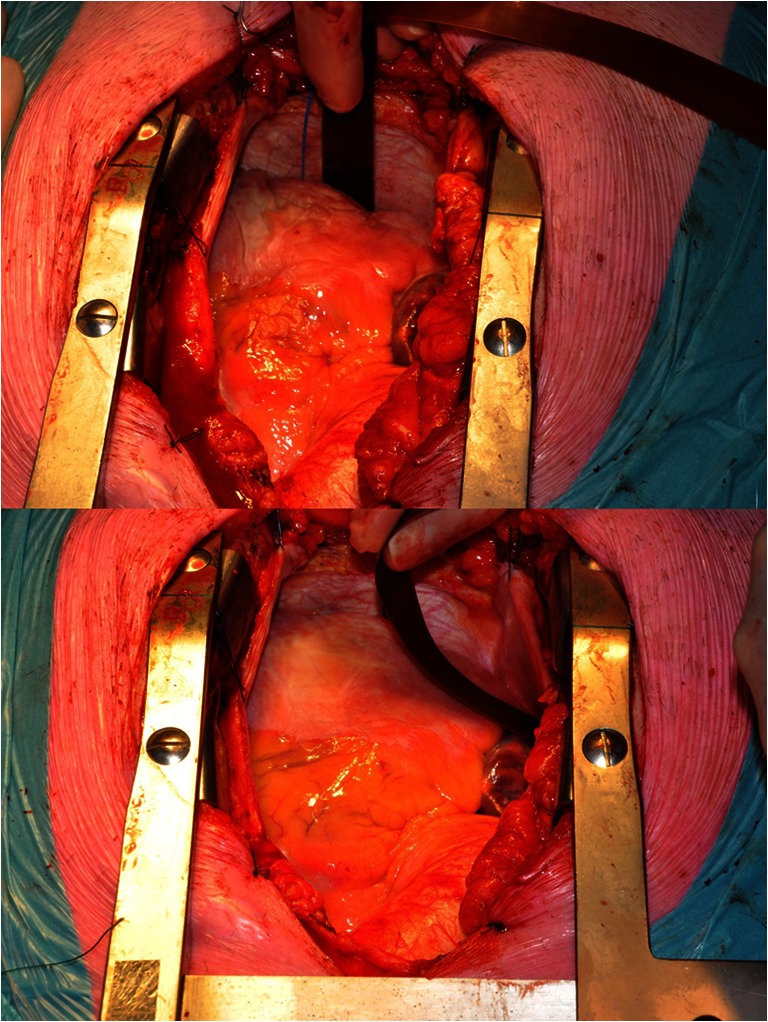
The electrode array placed between the pulmonary veins in the oblique sinus (*top*) and on the right atrial wall (*bottom*) during recording of epicardial electrograms

**Fig. 4:**
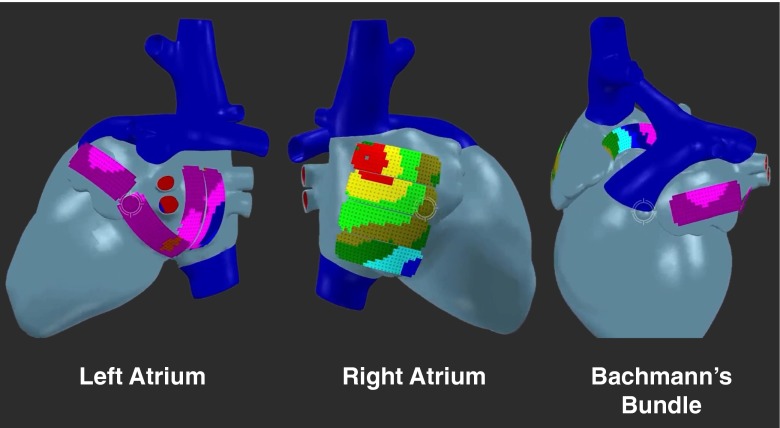
Mapping scheme demonstrating positions of the 192-electrode array on a 3D model. The left and right atria are covered by four positions each; to reach Bachmann’s bundle, the array is placed within the transverse sinus between the aorta and superior vena cava

Mapping is sequentially conducted along several imaginary lines between anatomical borders in order to cover the entire right and left atria (Fig. 3). The mapping array is shifted along these imaginary lines with a fixed orientation at each position visually trying to avoid omission of areas at the expense of possible overlap between successive mapping sites. Mapping of the right atrium starts at the cavotricuspid isthmus continuing up to the right atrial appendage, perpendicular to the caval veins. Bachmann’s bundle is mapped from the roof of the left atrium towards the superior cavo-atrial junction. For the left atrium, mapping is performed along the left atrioventricular groove from the lower border of the left inferior pulmonary vein towards the left atrial appendage and from the sinus oblique fold along the border of respectively the right and left pulmonary veins down to the atrioventricular groove. Figure 4 shows the positions of the 192-electrode array along these mapping lines. The mapping array is held in place through either light manual pressure or by means of the spatula. In case of the posterior area between the pulmonary veins, pressure from the weight of the heart and underlying structures provides stability for the mapping array. This technique was performed in 168 patients of 18 years and older, with coronary and/or structural heart disease, with or without AF, electively scheduled for cardiac surgery and a ventricular ejection fraction above 40 %. The mean duration of the entire mapping procedure including preparation time was 9 ± 2 min. Complications related to the mapping procedure during or after cardiac surgery were not observed.

### Mapping technique

The mapping array consists of an electroless nickel immersion gold (ENIG) plated electrode array, mounted on a thin, flexible DuPont™ Pyralux® copper-clad (25-μm thickness) polyimide laminate, and coverlay composite (25 μm) film (0.18 mm), manufactured by GS Swiss PCB AG, Küssnacht, Switzerland. Sterilization is performed by the local sterilization unit before use in the operation room. The sterilized array is connected to 3-meter-long, shielded flat cables delivered to the surgeon in a sterile sack. The flat cables are connected to a battery-driven, custom computerized mapping system which is connected to a laptop computer. Custom-made software visualizes real-time atrial electrograms recorded at all 192 electrodes to secure atrial recordings with good electrogram quality. Three channels are designated to display the surface ECG, reference signal and a calibration signal of 1-mV amplitude and 1000-ms pulse width. Five seconds of sinus rhythm and 10 s of AF are recorded at every mapping site. All recordings are amplified (gain 1000), filtered (bandwidth 0.5–400 Hz), sampled (1 kHz), analogue to digital converted (16 bits) and automatically stored on hard disk as E01-files labelled with the atrial rhythm, mapping site and patient identification code. Analysis will be performed offline as previously described [1].

## Discussion

Previous AF mapping studies during surgery did not cover the entire surface of both atria or used low-resolution arrays or recordings of short durations [2, 3]. Our mapping approach is the first high-resolution, multi-site mapping approach. It consists of a fixed mapping scheme with a 192-electrode mapping array, which results in a minimum of 1728 recording sites. The mapping array covers large areas of atrial tissue at each position resulting in a short all-over procedure time of 9 min without increasing cardiopulmonary bypass time. Unfortunately, high-resolution multi-site mapping is not performed simultaneously as this is technically not yet possible. However, with the reference signal from the right atrial free wall, we are able to construct a timeline between the different recordings and create an overall conduction map during sinus rhythm. This map can also demonstrate the possible overlap between recording sites. During AF, the reference signal obtains information about the changes in AF cycle length in order to assess the consistency of the arrhythmia. Previously, the indifferent electrode was fixed to the sternum; however, electrogram quality improved when the indifferent electrode was fixed to subcutaneous tissue. Mapping studies were only performed in patients undergoing elective cardiac surgery for the first time as mapping is technically unfeasible with prior cardiac surgery or pericardiocentesis due to pericardial scarring limiting access to atrial sites.

An advantage of epicardial mapping is that some potential arrhythmogenic structures such as Bachmann’s bundle can only be reached from the epicardial site and not from the endocardium. However, the interatrial septum cannot be mapped using a closed beating heart approach and the electrode array does not cover the myocardial sleeves within the pulmonary veins. In recent mapping studies of AF [1, 4], we introduced a so-called wavemapping technique to classify and quantify electrophysiological properties of fibrillation waves. This unique mapping approach makes it possible to study features of AF both in the spatial domain, like focal fibrillation waves or areas of conduction block, and the temporal domain, like irregularity of fibrillation intervals.

**Fig. 5:**
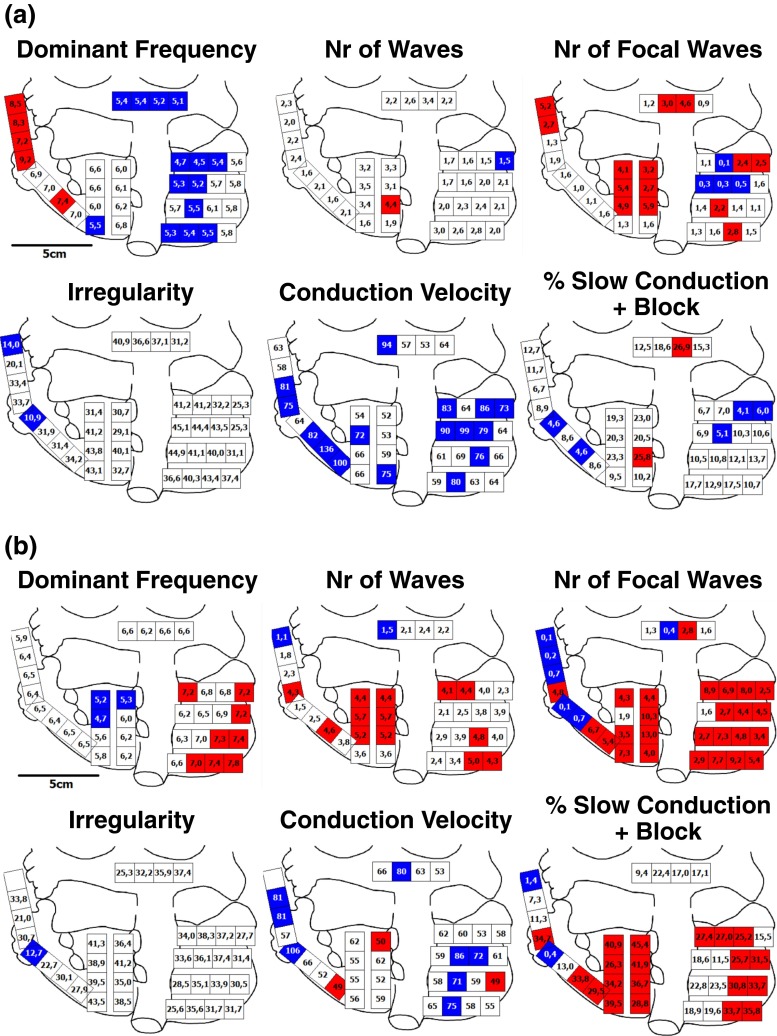
Mapping of induced vs long-standing persistent AF. Intra-atrial variation in various electrophysiological parameters measured during 10 s of AF, including dominant frequency (*blue* <6 Hz, *white* 6–7 Hz, *red* >7 Hz), number of fibrillation waves (*blue*<2 /cm^2^/s, *white* 3–4 /cm^2^/s, *red* >3 /cm^2^/s), number of focal waves (*blue* <1 /cm^2^/s, *white* 1–4 /cm^2^/s, *red* >4 /cm^2^/s), irregularity (*blue* <20 ms, *white* 20–50 ms, *red* >50 ms), conduction velocity (*blue* ≥70 cm/s, *white* 51–69 cm/s, *red* ≤50 cm/s) and incidence of slow conduction and block (*red* >25 %, *white* 6–25 %, *blue* ≤6 %). See text for further description. **a** Mapping of induced AF in a patient with coronary artery disease. **b** Mapping of long-standing persistent AF in a patient with mitral valve insufficiency

As an example, intra-atrial variation of various electrophysiological parameters, assessed during 10 s of induced AF in a patient with coronary artery disease, is summarized in Fig. 5a. Using specific cut-off values, every quadrant was depicted as blue (low), white (intermediate) or red (high).

The dominant frequency was 6.1 Hz and ranged from 4.5 to 9.2 Hz. The number of fibrillation and epicardial focal waves was on average respectively 2.3 and 2.1/cm^2^/s, varying between 1.5 to 4.4 /cm^2^/s and 0.1 to 5.9 /cm^2^/s. The averaged beat-to-beat irregularity was 35.0 ms and the averaged conduction velocity 72.2 cm/s; the degree of conduction delay and block during AF varied from 4.1 to 26.9 % (on average 12.6 %).

In comparison, electrophysiological parameters quantified during long-standing persistent AF in patient with mitral valve disease are demonstrated in Fig. 5b. The dominant frequency was 6.7 Hz (range, 4.7 to 7.8). Compared to all quadrants in the patient with coronary artery disease, the number of fibrillation and epicardial focal waves was considerably higher (number of fibrillation waves, 3.5 ± 1.3 /cm^2^/s (1.1–5.7), *P* < 0.001; number of focal waves, 4.3 ± 3.2 /cm^2^/s (0.1–13.0), *P* < 0.001). The averaged beat-to-beat irregularity was comparable (32.9 ms). Conduction velocity was on average 63 cm/s (*P* < 0.01), and the degree of conduction delay and block varied from 0.4 to 45.4 % (on average 24.8 %, *P* < 0.001). Hence, during long-standing persistent AF, the number of fibrillation waves and incidence of focal waves was higher and conduction abnormalities occurred more frequently; the areas involved are shown in the quadrant maps.

Therefore, this mapping technique can potentially identify vulnerable areas responsible for initiation and persistence of AF by localizing and quantifying the degree of electropathology in the individual patient. Furthermore, by understanding the electropathological substrate in AF patients and providing individualized diagnoses, high-resolution mapping might be able to direct current AF therapies more efficiently or may lead to new insights for treatment strategies which could in turn improve current treatment outcomes.
